# Effects of preconditioning with TNFα and IFNγ in angiogenic potential of mesenchymal stromal cell-derived extracellular vesicles

**DOI:** 10.3389/fcell.2023.1291016

**Published:** 2023-12-21

**Authors:** Sophie Cavallero, Samir Dekali, Nathalie Guitard, Héléne Théry, Carole Hélissey, Sabine François

**Affiliations:** ^1^ Armed Forces Biomedical Research Institute (IRBA), Department of Biological Effects of Radiation, Radiobiology Unit, Brétigny-sur-Orge, France; ^2^ Armed Forces Biomedical Research Institute (IRBA), Department of Biological Effects of Radiation, Emerging Technologies Risk Unit, Brétigny-sur-Orge, France; ^3^ Clinical Unit Research, HIA Begin, Paris, France

**Keywords:** mesenchymal stromal cells, extracellular vesicles, preconditioning, angiogenesis, interferon-gamma, tumor necrosis factor-alpha

## Abstract

**Introduction:** Mesenchymal stromal cells (MSCs) have demonstrated therapeutic properties both *in vitro* and *in vivo* to treat various diseases, including anti-inflammatory, immunomodulatory and pro-angiogenic effects. These therapeutic effects are mediated by their secretome composed of soluble factors and extracellular vesicles (EVs). The composition of EVs reflects the molecular and functional characteristics of parental cells. MSC preconditioning can alter the composition of EVs, thereby influencing their therapeutic potential.

**Methods:** MSCs were subjected to preconditioning with two cytokines, TNFα and IFNγ. Following 24 h of preconditioning, MSC-EVs secreted into the culture supernatant were isolated through tangential filtration. Particle concentration and size distribution were measured by nanoparticle tracking analysis, and the surface antigen expression of the EV-specific CD63 was quantified via Enzyme Linked ImmunoSorbent Assay. The angiogenic potential of MSCEVs obtained after preconditioning MSCs was assessed by the analysis of their protein composition and their influence on human umbilical vein endothelial cell (HUVECs) proliferation, migration, and tube-forming ability.

**Results:** Preconditioning with TNFα and IFNγ did not influence the MSC-EV profile but did induce changes in their protein content. Indeed, the expression of pro-angiogenic proteins increased in EVs from preconditioned MSCs compared to EVs from no-preconditioned MSCs. EVs from preconditioned MSCs tend to stimulate HUVEC migration, proliferation and tubeforming ability. These observations imply the presence of a pro-angiogenic potential in EVs obtained after preconditioning of MSCs with TNFα and IFNγ.

**Discussion:** In conclusion, it appears that the pro-angiogenic potential of EVs is enhanced through preconditioning of MSCs with TNFα and IFNγ. The use of these MSCs-EVs in therapy would circumvent the limitations of current cell-based therapies. Indeed, the therapeutic potential of MSC-EVs presents an attractive strategy for exploiting the clinical benefits of MSC therapy. For example, in the field of regenerative medicine, the exploitation of cell-free therapy using highly pro-angiogenic MSC-EVs is of great interest.

## Introduction

Mesenchymal stromal cells (MSCs) derived from adult tissues are characterized by their low immunogenicity, high proliferation capacity, differentiation capabilities, and modulation of physiological processes such as inflammation, hematopoiesis, and angiogenesis (Uccelli et al., 2008; Bernardo and Fibbe, 2013). They are currently considered an important therapeutic tool in neurological disorders, pulmonary dysfunctions, metabolic/endocrine-related diseases, reproductive disorders, skin burns, and cardiovascular conditions (Aggarwal and Pittenger, 2005; DiMarino et al., 2013; Vasanthan et al., 2020; Samsonraj et al., 2017; Wang et al., 2018). Moreover, due to their strong immunosuppressive capabilities, MSCs have been utilized in the treatment of immune-related disorders such as graft-versus-host disease, systemic lupus erythematosus, and Crohn’s disease and in severe accidental radiation burn (Le Blanc et al., 2008; Chen et al., 2019; Bey et al., 2007; Lataillade et al., 2007). Nonetheless, a primary constraint associated with employing MSCs for therapy resides in the invasive character of the cell collection procedures and the necessity for multiple doses to sustain the therapeutic impact. Additionally, the manipulation of MSCs can also lead to loss of cellular functionality and genetic instability when performed outside their natural niches (Sohni and Verfaillie, 2013). Indeed, injected MSCs into a given tissue or organ can cause vascular occlusion, an undesirable inflammatory response, transmission of the human pathogens, cardiac arrhythmia and even tumor formation (Arango-Rodriguez et al., 2015).

Numerous studies have demonstrated that the therapeutic efficacy of MSCs primarily relies on their paracrine activities. Indeed, the therapeutic functions of MSCs are facilitated through cell-cell interactions and/or the release of bioactive substances. In particular, the secretome derived from MSCs plays a crucial role in modulating various phases of the angiogenic process, stimulating endothelial cell functions *in vitro*, and promoting angiogenesis *in vivo* (Ulpiano et al., 2021). The MSCs secretome comprises a wide range of soluble protein factors, including growth factors and cytokines, and extracellular vesicles (EVs). EVs are nanoscale vesicles enclosed by the nuclear membrane, with diameters ranging from 40 to 150 nm, and they encompass a multitude of proteins, lipids, messenger RNAs (mRNAs), and regulatory microRNAs (miRNAs) (Cavallero et al., 2020). EVs play a pivotal role in mediating intercellular communication and interactions within biological systems. These membranous structures serve as essential messengers in various physiological and pathological processes (Liu and Wang, 2023) This intricate dependency results from a finely regulated sorting and selection process, enabling EVs to carry specific molecules based on the needs of the donor cell, the surrounding environment, and the potential recipient (Phinney and Pittenger, 2017).

In the literature, it is well-established that the functionality of MSCs can be altered through the manipulation of cell culture conditions, a procedure known as “preconditioning.” This approach prepares the cells for the specific *in vivo* environment in which they will be injected, thereby enhancing their therapeutic efficacy for a specific pathological condition (Hu and Li, 2018). For instance, hypoxia preconditioned MSCs have demonstrated efficacy as an allogeneic transplantation cell therapy, showing potential in mitigating renal fibrosis and inflammation (Ishiuchi et al., 2020) and improving both cellular survival and function (Elabd et al., 2018). Similarly, inflammatory preconditioning increases the immunomodulatory properties of MSCs *in vitro* and *in vivo* settings (Sarsenova et al., 2022). Utilizing *ex vivo* preconditioning approaches, which reproduce the injured environment through controlled culture conditions, offers a promising strategy to further enhance the therapeutic potential of MSCs (Hu and Li, 2018).

Preconditioning MSCs with inflammatory cytokines, such TNFα and IFNγ has been proven to be an effective approach for enhancing their regenerative capabilities (Gómez-Ferrer et al., 2021; Watanabe et al., 2022). However, the precise effects of this preconditioning on the protein content, and with a specific focus on the angiogenic potential of MSC-derived extracellular vesicles (MSC-EVs) remains poorly understood. Previous studies have mainly focused on the effects of preconditioning with hypoxia (Ferreira et al., 2018), and there is currently a lack of data regarding the specific changes induced by inflammatory cytokines in the protein composition of MSC-EVs. Therefore, investigating the effects of preconditioning on MSC-EVs could potentially open innovative therapeutic possibilities. By understanding the modifications in protein cargo induced by inflammatory cytokines more precisely, we can gain valuable insights into the mechanisms through which EVs can enhance their regenerative therapeutic potential. Consequently, a comprehensive understanding of the secretory activity of MSCs, in conjunction with their *in vivo* behavior and paracrine effects, is imperative for harnessing their clinical potential.

In the present study, we evaluated the influence of preconditioning MSCs with inflammatory stimuli (TNFα and IFNγ) on the protein profile and pro-angiogenic potential of MSC-EVs. Our results revealed that this preconditioning of MSCs led to the release of pro-angiogenic proteins by MSC-EVs. These vesicles were observed to exert an influence on the proliferation, migration and angiogenesis of human umbilical vein endothelial cells (HUVECs).

## Materials and methods

### Mesenchymal stem/stromal cells (MSCs) culture and characterization

Human mesenchymal stem/stromal cells (MSCs) were isolated from bone marrow aspirates, obtained from Lonza (Walkersville, MD, United States, WV IM-105), and were sourced from healthy informed consent donors aged 30–40 years with a BMI <25 and no history of diabetes. Unprocessed human bone marrow contained an average of 20 × 10^6^ nucleated cells/mL. The cells were seeded at a density of 0.16 × 10^6^ cell/cm^2^ in mesenchymal stem cell growth medium consisting of MEM Alpha Medium (Gibco ref. 22561-021), supplemented with Amphotericin B at 250 μg/mL (ref. Gibco 041-95780), 1% L-glutamine at 200 mM 100× (ref. Gibco 25030-024), 1% Penicillin-Streptomycin at 10,000 U/mL, 100× (ref. Gibco15140122) and 10% fetal bovine serum (Hyclone research grade fetal bovine serum, GE Healthcare Life sciences, ref. SV30160.03). The cultures were maintained at 37°C, 5% CO_2_, and 95% humidity. After 24 h, non-adherent cells were removed, and the medium was refreshed with mesenchymal stem cell growth medium. The culture medium was changed three times per week, until reaching 80% confluence, defining the cells as being at passage 0. Throughout each expansion phase, MSCs were seeded at a density of 5,000 cells per cm^2^. In this study, cells amplification was conducted up to passage 3 (P3), to minimize potential phenotypic alterations in the primary cells.

At P3, their differentiation potential and phenotype were analyzed following International Society for Cell and Gene Therapy (ISCT) recommendations (Horwitz et al., 2005; Viswanathan et al., 2019). For phenotyping, the cells were labeled with the CD90-FITC, CD105-FITC, CD73-PE, CD34-PE and CD45-PE antibodies (BD Biosciences) and analyzed by flow cytometry. To assess differentiation potential, MSCs were cultured with lineage-specific media: StemPro Adipogenesis Differentiation Kit (Gibco, A10070-01), StemPro Osteogenesis Differentiation Kit (Gibco, A10072-01), and StemPro Chondrogenesis Differentiation Kit (Gibco, A10071-01). The cells were incubated for 21 days to induce differentiation into adipocytes, osteocytes and chondrocytes. The differentiation outcomes were evaluated through staining with Oil Red O for adipocytes, alizarin red for osteocytes and alcian blue for chondrocytes. Observations were made using visible light microscopy (data shown in (Helissey et al., 2022)).

### Isolation and characterization of MSC-EVs

For preconditioning, at P3 of MSCs (80% of confluence), the cells were washed at least three times with sterile 1x PBS (Gibco) and were treated with 20 ng/mL Tumor Necrosis Factor alpha (TNFα) and 20 ng/mL Interferon gamma (IFNγ) for 24 h. Control MSCs (without preconditioning treatment) and TNFa/IFNγ preconditioned MSCs were washed in 1x PBS and cultured in serum free alpha MEM growth medium for 72 h. The culture medium was collected and centrifuged (3000g, 10min) to remove whole cells and debris. The resulting supernatant underwent tangential flow filtration (TFF) using disposable hollow fiber filters (Repligen, mPES 500k) to specifically isolate and concentrate MSC-derived extracellular vesicles (MSC-EVs).

The isolated MSC-EVs were then characterized according to the minimal information for studies of extracellular vesicles (MISEV) guidelines established by International Society for Extracellular Vesicles (ISEV) (Théry et al., 2018; Witwer et al., 2021). Quantification and sizing of particles present in the samples were performed using the NanoSight NS300 instrument (Malvern Panalytical). CD63 levels, widely used as EVs markers, were measured in the sample using the ExoELISA-ULTRA CD63 kit (System Biosciences, California, CA, United States) according to the manufacturer’s protocol.

### Analysis of angiogenesis protein expression in MSC-EVs

#### Proteome profiler

Protein concentration of MSC-EVs samples were measured using a BCA protein assay kit (Thermo Scientific Pierce, Rockford, IL, United States). Protein expression was analyzed using Proteome Profiler Human Angiogenesis Array kit (R&D Systems, catalogue number ARY007). The Proteome Profiler Human Angiogenesis Array Kit is a membrane-based sandwich immunoassay. Samples are mixed with a cocktail of biotinylated detection antibodies (Step 1) and then incubated with the array membrane, which is spotted in duplicate with capture antibodies to specific target proteins (Step 2). Captured proteins are visualized using chemiluminescent detection reagents (Step 3). The signal produced is proportional to the amount of bound analyte and was detected using a ChemiDoc XRS + System (BioRad, California, CA, United States). The signal density of each blot was quantified by dot blot analysis on NIH ImageJ analysis software 1.8.0.

#### Enzyme-linked immuno assay

The level of three pro-angiogenic proteins was measured with a Quantikine ELISA kit (R&D systems): IL8 (D8000C), MCP-1 (DCP00) and CXCL16 (DCX160), following the manufacturer’s instructions.

### Analysis of influence of MSC-EVs on HUVECs function

#### HUVECs proliferation analysis

To measure the impact of MSC-EVs samples on HUVECs proliferation, HUVECs were cultured for 24 h in LSGS-supplemented Medium 200 (Gibco) with MSC-EVs samples. The cells were then trypsinized and 10 µL of cell suspension was mixed with trypan blue. Viable cells were counted automatically using automated cell counter (Luna II, Logos Biosystems).

#### HUVECs migration analysis

HUVECs migration was analyzed using xCELLigence real-time cell analysis (RTCA). Cell migration measurements were carried-out using 16-well CIM-plates (Agilent, Reference 5665817001). CIM-plates have upper and lower chambers for each well separated by a microporous membrane (in polyethylene terephthalate) with pore size of 8 µm and in contact with microelectrodes. Briefly, 160 µL per well of LSGS-supplemented Medium 200 (Gibco) were deposited in lower chambers. CIM-plates were then assembled under the cell culture hood using CIM-Plate 16 assembly tool. Subsequently, 25 µL/well of LSGS-supplemented Medium 200 (Gibco) were added in upper chambers to cover the membrane surface, and CIM-Plates were incubated for 1 hour at 37°C, 95% humidity and 5% CO_2_. Background measurement step was then performed (every minute during 10 min). HUVECs cell suspensions (100 μL and 1 × 10^4^ viable cells/well) containing or not EVs (4 × 10^7^ EV/well) were then added in upper chambers. Each condition was performed in duplicates with a programmed signal detection schedule once every 10 s during 24 h (early effects), then every 10 min for 48 h (late effects). Control cells were only incubated with LSGS-supplemented Medium 200 (Gibco). Results are expressed using normalized cell index (NCI) as described by Bourgois et al. (2019).

#### *In Vitro* tube formation assay

Geltrex LDEV-Free Reduced Growth Factor Basement Membrane Matrix (Gibco, 100 µL/well) was coated onto 24-well plates and cultured in a 37°C for 30 min to allow matrix gel polymerization. HUVECs (Gibco) were removed from culture after 7 days in LSGS-supplemented Medium 200 (Gibco), trypsinized and resuspended in LSGS-supplemented Medium 200. HUVECs (5 × 10^4^ cell/well) were seeded into each well in LSGS-supplemented Medium 200 with MSC-EVs samples (2 × 10^8^ EV/well). After 12 h incubation, the cells were stained with 2 μg/mL of Calcein, AM (Invitrogen, Waltham, MA, United States) incubated for 30 min at 37°C. Fluorescence images were captured at a ×4 magnification using a CKX53 brightfield and epifluorescence microscope (Olympus, Allentown, PA, United States). The quantification of angiogenic network formation was performed by counting the number of nodes, junctions, segments and calculating the total segment length based on the collected images, utilizing the NIH ImageJ analysis software, as described in (Gilles et al., 2012).

### Statistical analysis

For statistical analysis of the results, the GraphPad Prism 7.0 software (https://graphpad-prism.software.informer.com/7.0/) was used. The Mann–Whitney test was applied to assess statistical significance, with significance levels established at *p* < 0.05 (*), *p* < 0.01 (**), and *p* < 0.001 (***). All values were presented as the mean ± standard error of the mean (SEM).

## Results

### Effect of preconditioning with TNFα and IFNγ on profiles of MSC-EVs

**FIGURE 1:**
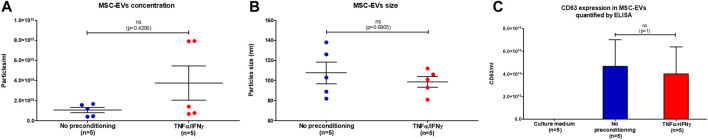
MSC-EVs characterization. **(A)** Concentration of particles present in MSC-EVs samples measured by NTA, graph showing the calculated mean ± SEM (Mann-Whitney test, *p*=0,4206) **(B)** Size of particles present in MSC-EVs samples measured by NTA, graph showing the calculated mean ± SEM (Mann-Whitney test, *p*=0,6905) **(C)** Quantification of CD63 expression, marker of extracellular vesicles, in MSC-EVs samples and in culture medium measured by ELISA, graph showing the calculated mean ± SEM (Mann-Whitney test, *p*=1).

The size and concentration of extracellular vesicles in MSC-EVs samples were measured by Nanoparticle Tracking Analysis (NTA). The NTA results revealed no significant difference in the concentration of EVs (Mann–Whitney test, *p* = 0.4206) and in vesicle size (Mann–Whitney test, *p* = 0.6905) between samples of MSC-EVs (Figures 1A, B). The mean concentration of particles measured by NTA was 1.056 × 10^9^/mL for EVs from no-preconditioned MSCs and 3.741 × 10^9^/ml for EVs from preconditioned MSCs. The mean particle size of EVs from preconditioned MSCs was 99 nm and 108 nm for EVs from no-preconditioned MSCs. The level of CD63, a specific marker for EVs, in MSC-EVs samples were measured by ELISA (Figure 1C). The mean concentration of CD63 was close in between MSC-EVs samples (Mann Whitney test, *p* = 1), 46.55 × 10^9^/ml in EVs from no-preconditioned MSCs and 39.98 × 10^9^/ml in EVs from preconditioned MSCs. The absence of CD63 expression in the culture medium shows the specificity of CD63 for EVs. These results indicate that preconditioning of MSCs with TNFα and IFNγ did not affect the characterization criteria of MSC-EVs. However, as illustrated in Figure 1A, the concentration of MSC-EVs after preconditioning is heterogeneous, with some samples showing an increase by a factor of 4.

### Effect of preconditioning with TNFα and IFNγ on the angiogenesis protein expression of MSC-EVs

**FIGURE 2:**
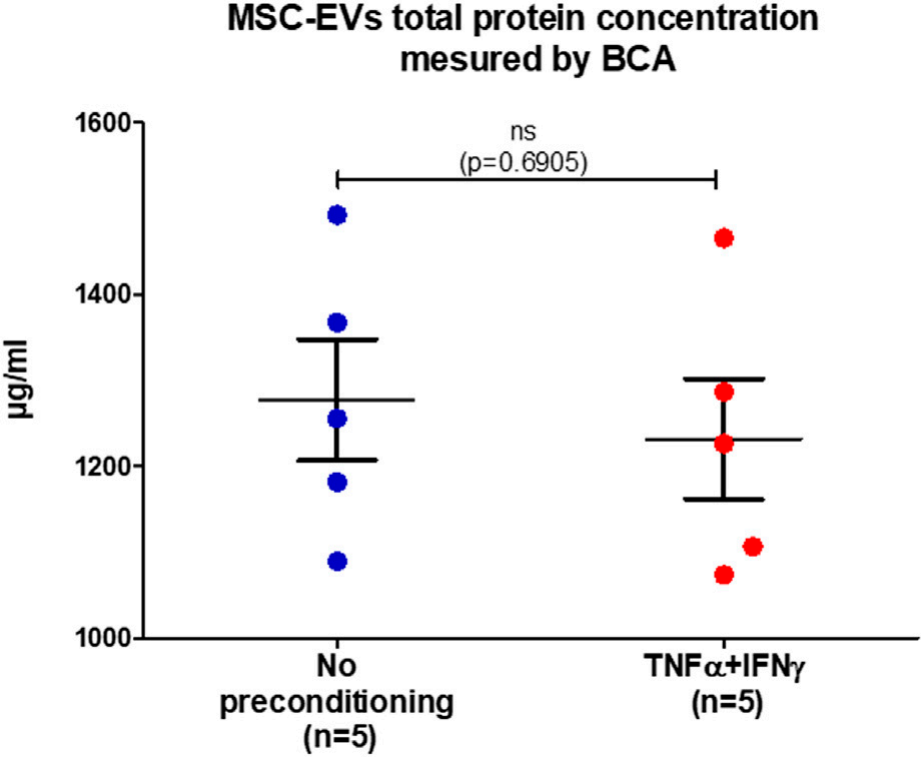
MSC-EVs total protein concentration. Concentration of total protein present in MSC-EVs samples measured by BCA, graph showing the calculated mean ± SEM. (Mann-Whitney test, *p*=0.6905).

Total protein concentration in MSC-EVs samples was measured by BCA (Bicinchoninic acid assay) (Figure 2). Both samples showed equivalent results, a similarity verified by performing the Mann-Whitney test, resulting in a *p*-value of 0.6905. In EVs from no-preconditioned MSCs, the mean total protein concentration of protein total was 1,278 μg/mL and in EVs from preconditioned MSCs, it was 1,232 μg/mL. This result indicates that the preconditioning of MSCs with IFNγ and TNFα has no effect on the total protein concentration within MSC-EVs. The absence of correlation between the quantity of EVs and their total protein content indicates that there is no linear or predictable relationship between these two parameters (r = 0.5153, *p* = 0.1557). These observations highlight a level of complexity and heterogeneity in the composition of MSC-EVs.

**FIGURE 3:**
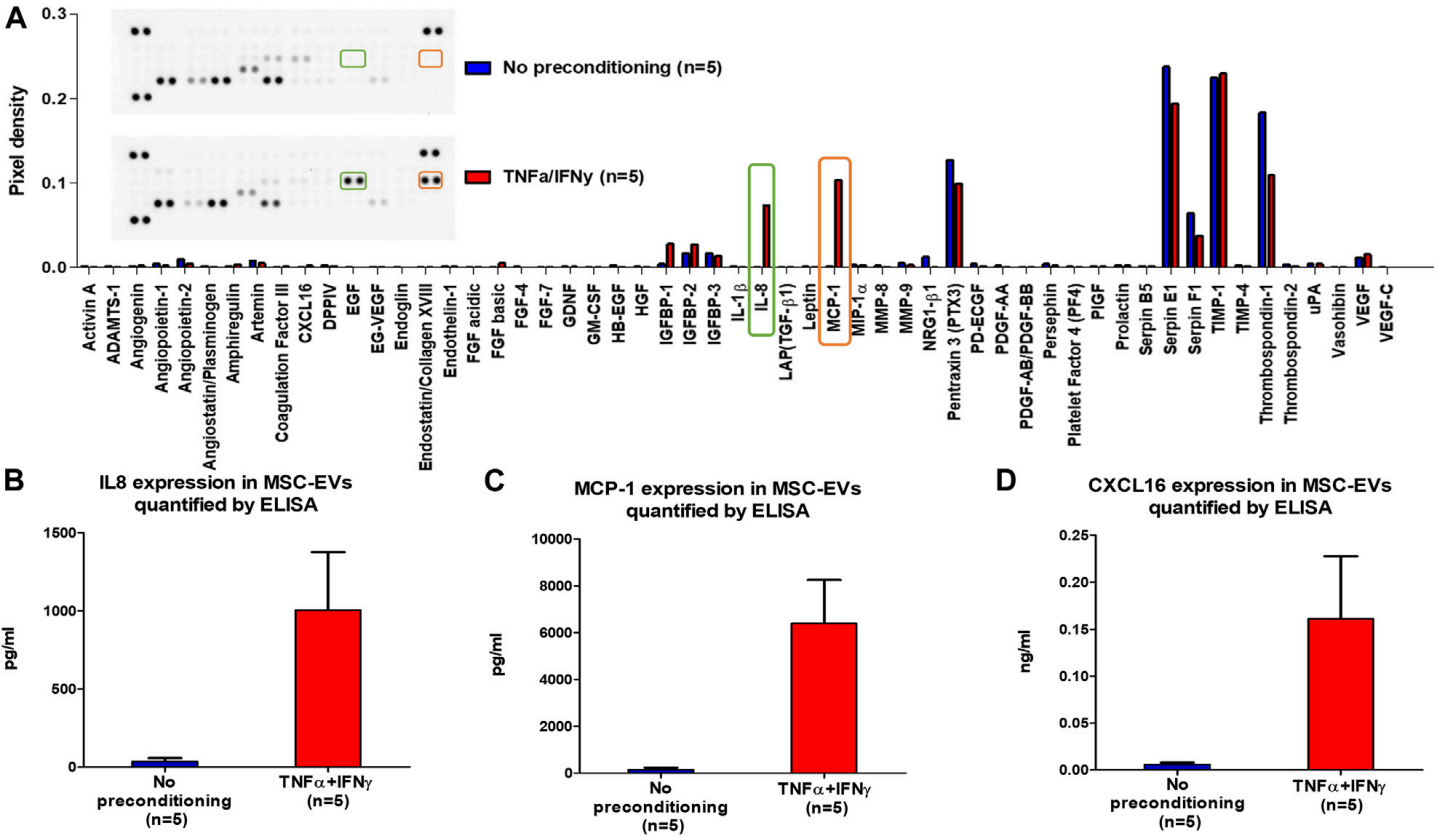
MSC-EVs angiogenesis protein expression. **(A)** Profiling of angiogenesis-regulating proteins secreted by MSC-EVs samples obtained with Proteome Profiler Human Angiogenesis Array Kit (R&D). The upper section displays a representative image of the dot blot, while the lower section exhibits a graph illustrating the pixel density of each protein’s dot blot signal, normalized to the total membrane signal **(B)** IL8 expression in MSC-EVs samples measured by ELISA (mean ± SEM) **(C)** MCP-1 expression in MSC-EVs samples measured by ELISA (mean ± SEM) **(D)** CXCL16 expression in MSC-EVs samples measured by ELISA (mean ± SEM).

To assess the impact of MSCs preconditioning on the expression of angiogenic proteins in MSC-EVs, the expression of 55 angiogenesis related proteins was analyzed in MSC-EVs samples using the Proteome Profiler Human Angiogenesis Array Kit (Figure 3). This semi-quantitative analysis seemed to show apparent differences in the expression of some proteins between EVs from preconditioned or no-preconditioned MSCs. Notably, an increase in the expression of pro-angiogenic proteins (IL8, MCP-1, … ) and a decrease in the expression of anti-angiogenic proteins (Thrombospondin-1, … ) appeared to occur in EVs from preconditioned MSCs. ELISA quantification further confirmed the significant overexpression of three proteins essential to angiogenesis, IL-8, MCP1, and CXCL16 in EVs from preconditioned MSCs (Figures 3B–D). These results suggest that preconditioning with TNFα and IFNγ stimulated the expression of pro-angiogenic proteins in MSC-EVs.

### Effect of preconditioning MSC-EV with TNFα and IFNγ on angiogenesis

Angiogenesis requires numerous processes, including vascular endothelial cell proliferation, migration and differentiation. The angiogenic potential of MSC-EVs following preconditioning was evaluated using human umbilical vein endothelial cells (HUVECs).

**FIGURE 4:**
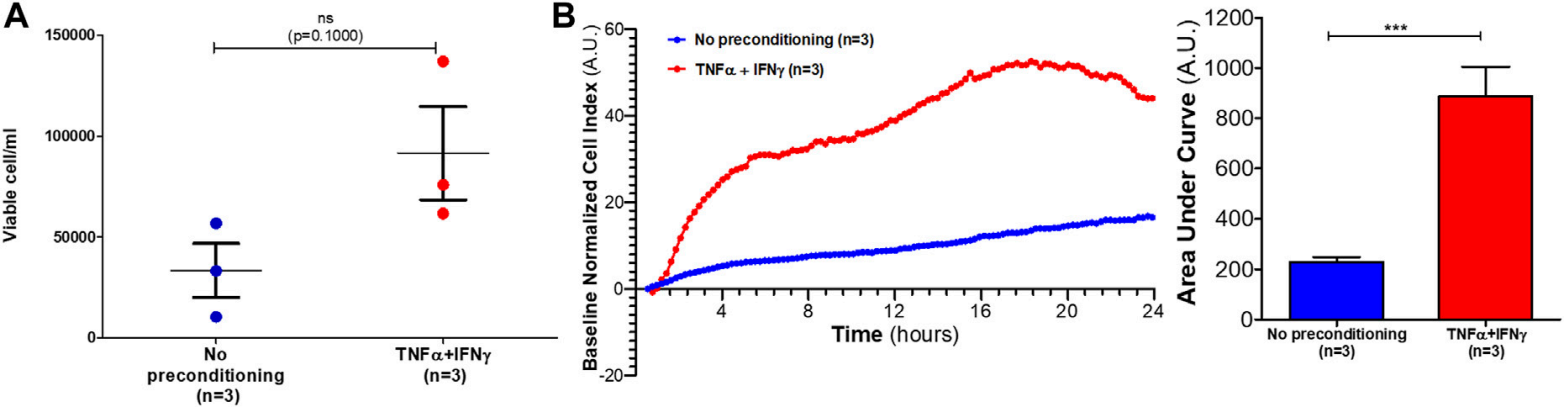
Effects of MSC-EVs samples on HUVECs proliferation and migration. **(A)** HUVECs concentration was measured after 24 h incubation with MSC-EVs sample (mean ± SEM) **(B)** HUVECs migration incubated with MSC-EVs samples was measured for 24 h using xCELLigence RTCA DP. In left graph, each curve represents mean of three independent experiments of the baseline normalized cell index expressed in arbitrary units (A.U.). Areas under curves were calculated after each treatment (expressed in A.U.) and mean ± SEM represented on right graph (Mann-Whitney test, ***p = 0,0002).

To evaluate the proliferation response of HUVECs to EVs from preconditioned MSCs, HUVECs were incubated with MSC-EVs samples. After 24 h, viable cells concentration was measured. HUVECs concentration tended to increase after 24 h of incubation with EVs from preconditioned MSCs (mean of viable cell: 3 × 10^4^ cell/mL) as compared of incubation with EVs from no-preconditioned MSCs (mean of viable cell: 9 × 104 cell/mL) (Figure 4A). However, Mann-Whitney tests performed on samples did not show significant differences between the two conditions (*p* = 0.1000).

A migration assay using xCELLigence real-time cell analyzer was performed to evaluate the pro-migratory effects of EVs from preconditioned MSCs. After 24 h incubation with EVs from preconditioned MSCs, HUVECs baseline normalized cell index significantly increased as compared to incubation with EVs from no-preconditioned MSCs (*p* = 0.0002) (Figure 4B). Therefore, EVs from preconditioned MSCs significantly increased HUVECs migration compared to no-preconditioned EVs treatment.

**FIGURE 5:**
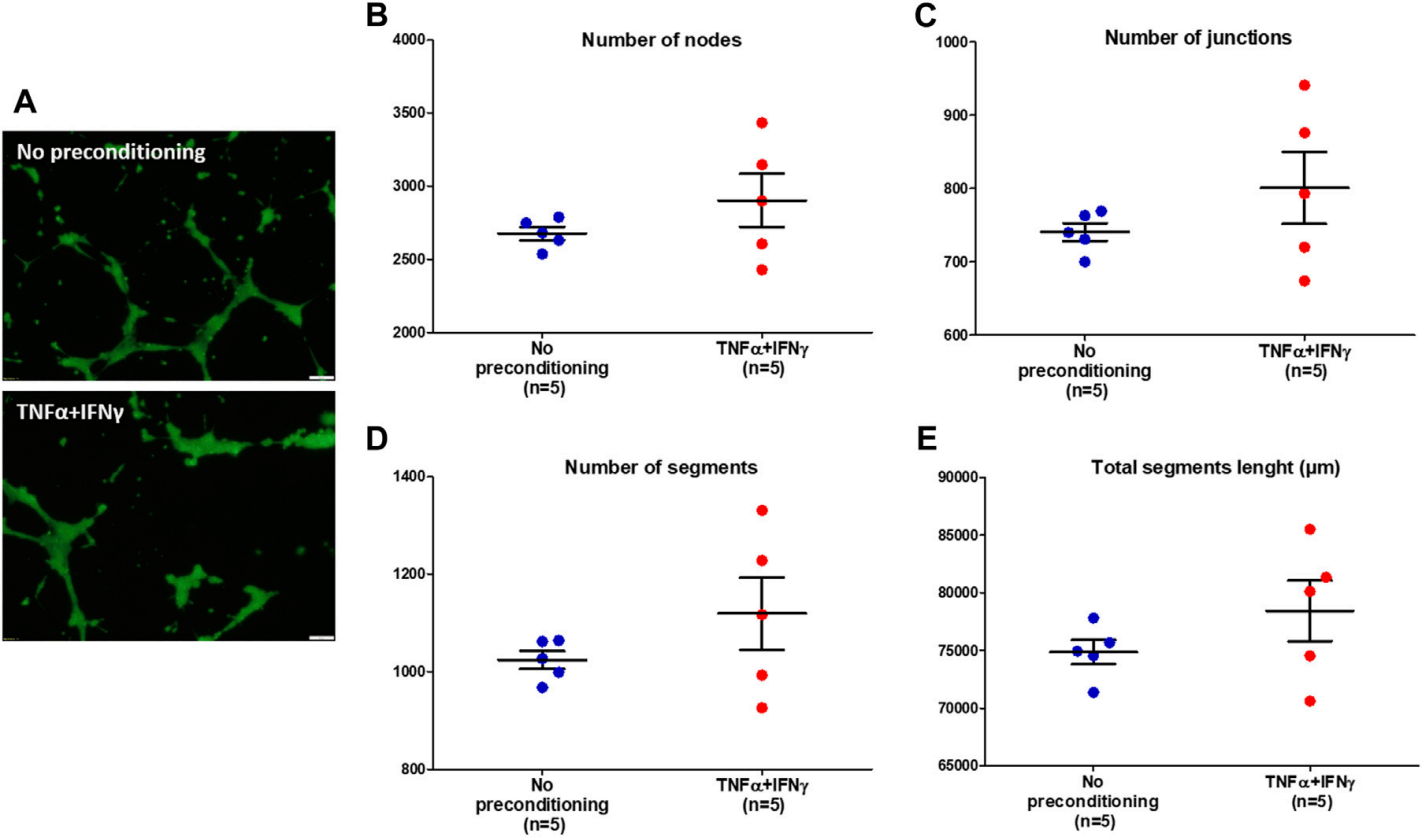
Tube formation of HUVECs incubated with MSC-EVs samples. **(A)** Representative images (×10 magnification, scale bar = 50 µm) of tube formation by HUVECs after incubation with MSC-EVs samples, taken by fluorescence microscopy (calcein-AM staining), 12 h after cell seeding. **(B)** Number of nodes quantified under different experimental conditions using ImageJ software **(C)** Number of junctions quantified under different experimental conditions using ImageJ software **(D)** Number of segments quantified under different experimental conditions using ImageJ software **(E)** Total segments length quantified under different experimental conditions using ImageJ software.

To investigate the effects of EVs from preconditioned MSCs on the ability of HUVECs to form tubule networks *in vitro*, a tube formation assay was performed. After 12 h incubation in the presence of MSC-EVs samples, HUVECs cultured on a basement membrane extract matrix differentiated and formed tubular structures (Figure 5A). The network of tubes formed was quantified with the NIH ImageJ analysis software. Four indicators to determine the angiogenic effects of MSC-EVs samples were analyzed: the number of nodes (Figure 5B), the number of junctions (Figure 5C), the number of segments (Figure 5D) and total segments length (Figure 5E). After incubation of HUVECs in the presence of EVs from preconditioned MSCs, the number of nodes, junctions, and segments as well as total segment length appeared to increase compared to HUVECs incubated in the presence of EVs from no-preconditioned MSCs. However, Mann-Whitney tests performed on the samples showed no significant differences between the two conditions (*p* = 0.5476 for number of nodes, junction and segments, *p* = 0.4206 for total segments lenght). Nevertheless, in 50% of the *in vitro* neovascularization assays conducted with EVs from preconditioned MSC, we observed an increase in the number of nodes, tubes, and in total segment length.

Taken together, these results suggest pro-angiogenic potential in preconditioned MSC-derived EVs. But these vesicles appear to be too heterogeneous and in insufficient quantity to elicit significant stimulation.

## Discussion

MSCs have been shown to have therapeutic properties *in vitro* and *in vivo*, including anti-inflammatory and immunomodulatory effects (Bernardo and Fibbe, 2013). MSCs have also been shown to promote angiogenesis and accelerate tissue healing (Caplan and Correa, 2011). All these properties make MSCs the most frequently used cell type in therapy. Indeed, the accumulation of preclinical evidence on the promising potential of MSC-based cell therapy has allowed its transposition into clinical applications (García-Bernal et al., 2021). However, despite several years of use of MSCs as therapeutic agents, some aspects of this therapy warrant consideration (Kou et al., 2022). First of all, the proliferative capacity of MSCs is gradually weakened and is accompanied by a certain degree of differentiation which impacts their regulatory and therapeutic capacity (Neuhuber et al., 2008). Then, in the *in vivo* environment, the self-renewal capacity of MSCs cannot be controlled with a consequent tumorigenicity potential (Jeong et al., 2011). Moreover, although MSCs have a strong potential for regenerative regulation, it is uncertain whether they can target or remain at the damaged site after intravenous injection (Karp and Leng Teo, 2009). MSCs have also been found to cause and promote the growth of various types of cancer (Adamo et al., 2019). Additionally, there are the usual risks associated with cell therapy such as viral infection and immune rejection as well as storage and transportation issues (Ankrum and Karp, 2010).

The discovery that most of the therapeutic effects of MSCs depend on their paracrine action, in particular EVs, and that these vesicles can replace their parent cells offers interesting prospects for the development of innovative therapies (Jafarinia et al., 2020). EVs have numerous advantages (Kou et al., 2022): they lack self-replication capacity, significantly mitigating the risk of tumorigenicity (Phinney and Pittenger, 2017). In comparison to cell therapy, EVs present a safer alternative. As nanoparticles, they exhibit both biocompatibility and low immunogenicity, faciliting their traversal of protective barriers such as the blood-brain barrier (Milbank et al., 2021). They can be continuously secreted by immortalized cells to obtain enough (Xunian and Kalluri, 2020). EVs protect their internal biomolecular activity via their lipid membrane structure, can be stored for an extended period at −80°C and are not prone to deactivation even after repeated freezing and thawing (Zhuang et al., 2021; Watanabe et al., 2022) they possess an encapsulation capacity, enabling the loading of specific drugs for targeted transport to recipient cells (Herrmann et al., 2021).

The paracrine activity of MSCs is influenced by the cellular microenvironment. The composition and activity of MSC-EVs can therefore be modulated by the culture medium of the MSCs (Kusuma et al., 2017). Consequently, preconditioning of MSCs (i.e., intentionally exposing them to a controlled amount of stimulus for a defined period of time in order to produce a desired response) allow to influence the therapeutic potential of MSC-EVs (Baraniak and McDevitt, 2010). Studies have shown that preconditioning MSCs via soluble factors or O_2_ tension (hypoxia) or 3D culture or mechanical stimulation modulate the composition and activity of MSC-EVs (Kusuma et al., 2017). In a healthy organism, trauma triggers an inflammatory response, leading to the recruitment of inflammatory cells and the release of various cytokines at sites of injury, including TNFα, IFNγ, interleukin (IL)-1, IL-4 and IL-6 (Kany et al., 2019). Angiogenesis is essential for tissue repair, a robust vascular network is essential to supply blood and growth factors to injured tissues (Li et al., 2003a; Eelen et al., 2020). Thus, in this study, we examine the impact of creating an inflammatory environment, simulated by preconditioning MSCs with TNFα and IFNγ, on the angiogenic potential on MSC-EVs.

Many studies have shown that MSC-EVs secretion can be stimulated by preconditioning (Jin et al., 2022). Preconditioning with thrombin, hypoxia, H_2_O_2_ and LPS significantly enhances EV biogenesis and secretion compared with naive MSCs. Notably, preconditioning with thrombin can easily increase EV production by more than fourfold compared with other preconditioning regimens (Sung et al., 2019). Our inflammatory preconditioning of MSCs did not alter the size or concentration of the isolated MSC-EVs.

In our study, preconditioning with TNFα and IFNγ stimulated the secretion of pro-angiogenic proteins by MSC-EVs, notably IL8, MCP-1, and CXCL16. These proteins are highly pro-angiogenic. IL8 acts via CXCR2, a surface receptor for endothelial cells, to induce endothelial cell proliferation and capillary tube organization (Li et al., 2003b). MCP-1 is a potent angiogenic chemokine acting directly on endothelial cells to promote angiogenesis. The pro-angiogenic effect of MCP-1 is mediated by its promotion of endothelial cell migration (Wang et al., 2012). CXCL16 promotes angiogenesis via autocrine signaling in HUVECs, involving activation of the ERK1/2, Akt and p38 pathways and subsequent upregulation of HIF-1α. In addition, CXCL16 increases VEGF secretion in HUVECs, most likely via regulation of HIF-1α (Li et al., 2003b). Thus, our prior inflammatory preconditioning of MSCs appears to promote the production of MSC-EVs that activate endothelial cells in the angiogenesis process.

To confirm this hypothesis, we assessed the effect of EVs from preconditioned MSCs on HUVEC proliferation, migration and angiogenesis stimulation, which are key processes in angiogenesis. EVs from preconditioned MSC significantly stimulated HUVEC migration. They also tended to increase their proliferation and tube-forming ability. This disparity in efficacy may be attributed to the fact that the concentration of total proteins within MSC-EVs is not linearly correlated with the quantity of EVs. However, it is reasonable to suspect that the quantity of MSC-EVs used, as well as the heterogeneity of their protein concentrations, may impose limitations on *in vitro* angiogenesis. Nevertheless, it is worth noting that the majority of published studies examining the impact of EVs on *in vitro* angiogenesis do not provide specific details regarding the concentrations of EVs employed. In the subsequent phases of this study, we will explore different concentrations of MSC-EVs.

Preconditioning of MSCs with TNFα and IFNγ appears to enhance the pro-angiogenic potential of MSC-EVs. However, it is imperative to ascertain whether this preconditioning does not lead to the secretion of pro-inflammatory proteins, in order for it to be conducive to tissue repair. Moreover, a deeper understanding of the underlying mechanisms of the interaction between MSCs and EVs, as well as their modulation by pro-inflammatory conditions, will be essential to optimize MSC-EV-based therapy. While MSC-EVs therapy holds promising potential as an innovative “cell-free” therapeutic approach, there exist several obstacles that necessitate addressing before clinical application. Among these challenges, the standardization of MSC culture conditions and the formulation of protocols for the isolation, preservation, and storage of EVs stand out as pivotal steps. It is imperative to recognize that the release, biogenesis, and molecular composition of MSC-EVs are significantly impacted by the physical conditions of cell culture. Hence, there is an urgent requirement to thoroughly investigate their contents to attain EVs with exceptional uniformity, ultimately enhancing their specific therapeutic effectiveness.

## Conclusion

In this study, we observed that preconditioning MSCs with TNFα and IFNγ appears to promote a pro-angiogenic potential of MSC-EVs, thereby fostering the migration and proliferation of endothelial cells. These results indicate that preconditioning with inflammatory cytokines of MSCs can enhance the therapeutic potential of MSC-EVs. Indeed, in regenerative medicine, it would be interesting to benefit from an acellular therapy via MSC-EVs having in particular their pro-angiogenic potential strongly stimulated. However, as our study was limited to the cellular level, further research on animal models will be carried out to confirm the results obtained.

## Data Availability

The original contributions presented in the study are included in the article/supplementary material, further inquiries can be directed to the corresponding author.

## References

[B1] Adamo A. Dal Collo G. Bazzoni R. Krampera M. (2019). Role of mesenchymal stromal cell-derived extracellular vesicles in tumour microenvironment. Biochimica Biophysica Acta (BBA) - Rev. Cancer 1871 (1), 192–198. 10.1016/j.bbcan.2018.12.001 30599188

[B2] Aggarwal S. Pittenger M. F. (2005). Human mesenchymal stem cells modulate allogeneic immune cell responses. Blood 105 (4), 1815–1822. 10.1182/blood-2004-04-1559 15494428

[B3] Ankrum J. Karp J. M. (2010). Mesenchymal stem cell therapy: two steps forward, one step back. Trends Mol. Med. 16 (5), 203–209. 10.1016/j.molmed.2010.02.005 20335067 PMC2881950

[B4] Arango-Rodriguez M. L. Ezquer F. Ezquer M. Conget P. (2015). Could cancer and infection be adverse effects of mesenchymal stromal cell therapy? World J. Stem Cells 7 (2), 408–417. 10.4252/wjsc.v7.i2.408 25815124 PMC4369496

[B5] Baraniak P. R. McDevitt T. C. (2010). Stem cell paracrine actions and tissue regeneration. Regen. Med. 5 (1), 121–143. 10.2217/rme.09.74 20017699 PMC2833273

[B6] Bernardo M. E. Fibbe W. E. (2013). Mesenchymal stromal cells: sensors and switchers of inflammation. Cell Stem Cell 13 (4), 392–402. 10.1016/j.stem.2013.09.006 24094322

[B7] Bey E. Duhamel P. Lataillade J. J. de Revel T. Carsin H. Gourmelon P. (2007). Treatment of radiation burns with surgery and cell therapy. A report of two cases. Bull. Acad. Natl. Med. 191 (6), 971–978. discussion 979. French. 18402158

[B8] Bourgois A. Crouzier D. Legrand F. X. Raffin F. Boyard A. Girleanu M. (2019). Alumina nanoparticles size and crystalline phase impact on cytotoxic effect on alveolar epithelial cells after simple or HCl combined exposures. Toxicol Vitro 59, 135–149. 10.1016/j.tiv.2019.04.016 31004741

[B9] Caplan A. I. Correa D. (2011). The MSC: an injury drugstore. Cell Stem Cell 9 (1), 11–15. 10.1016/j.stem.2011.06.008 21726829 PMC3144500

[B10] Cavallero S. Riccobono D. Drouet M. François S. (2020). MSC-derived extracellular vesicles: new emergency treatment to limit the development of radiation-induced hematopoietic syndrome? Health Phys. 119 (1), 21–36. 10.1097/HP.0000000000001264 32384375

[B11] Chen Y. Yu Q. Hu Y. Shi Y. (2019). Current research and use of mesenchymal stem cells in the therapy of autoimmune diseases. Curr. Stem Cell Res. Ther. 14 (7), 579–582. 10.2174/1574888X14666190429141421 31729289

[B12] DiMarino A. M. Caplan A. I. Bonfield T. L. (2013). Mesenchymal stem cells in tissue repair. Front. Immunol. 4, 201. 10.3389/fimmu.2013.00201 24027567 PMC3761350

[B13] Eelen G. Treps L. Li X. Carmeliet P. (2020). Basic and therapeutic aspects of angiogenesis updated. Circ. Res. 127 (2), 310–329. 10.1161/CIRCRESAHA.120.316851 32833569

[B14] Elabd C. Ichim T. E. Miller K. Anneling A. Grinstein V. Vargas V. (2018). Comparing atmospheric and hypoxic cultured mesenchymal stem cell transcriptome: implication for stem cell therapies targeting intervertebral discs. J. Transl. Med. 16 (1), 222. 10.1186/s12967-018-1601-9 30097061 PMC6086019

[B15] Ferreira J. R. Teixeira G. Q. Santos S. G. Barbosa M. A. Almeida-Porada G. Gonçalves R. M. (2018). Mesenchymal stromal cell secretome: influencing therapeutic potential by cellular pre-conditioning. Front. Immunol. 9, 9. 10.3389/fimmu.2018.02837 30564236 PMC6288292

[B16] García-Bernal D. García-Arranz M. Yáñez R. M. Hervás-Salcedo R. Cortés A. Fernández-García M. (2021). The current status of mesenchymal stromal cells: controversies, unresolved issues and some promising solutions to improve their therapeutic efficacy. Front. Cell Dev. Biol. 9, 9. 10.3389/fcell.2021.650664 PMC8007911 33796536

[B17] Gilles C. Marianna M. Jose C. Ilaria C. (2012). “Angiogenesis analyzer for ImageJ,” in 4th ImageJ user and developer conference proceedings (Luxembourg: Mondorf-les-Bains), 198–201.

[B18] Gómez-Ferrer M. Amaro-Prellezo E. Dorronsoro A. Sánchez-Sánchez R. Vicente Á. Cosín-Roger J. (2021). HIF-overexpression and pro-inflammatory priming in human mesenchymal stromal cells improves the healing properties of extracellular vesicles in experimental crohn’s disease. Int. J. Mol. Sci. 22 (20), 11269. 10.3390/ijms222011269 34681929 PMC8540690

[B19] Helissey C. Guitard N. Théry H. Goulinet S. Mauduit P. Girleanu M. (2022). Two new potential therapeutic approaches in radiation cystitis derived from mesenchymal stem cells: extracellular vesicles and conditioned medium. Biol. (Basel) 11 (7), 980. 10.3390/biology11070980 PMC9312102 36101361

[B20] Herrmann I. K. Wood M. J. A. Fuhrmann G. (2021). Extracellular vesicles as a next-generation drug delivery platform. Nat. Nanotechnol. 16 (7), 748–759. 10.1038/s41565-021-00931-2 34211166

[B21] Horwitz E. M. Le Blanc K. Dominici M. Mueller I. Slaper-Cortenbach I. Marini F. C. (2005). Clarification of the nomenclature for MSC: the international society for cellular therapy position statement. Cytotherapy 7 (5), 393–395. 10.1080/14653240500319234 16236628

[B22] Hu C. Li L. (2018). Preconditioning influences mesenchymal stem cell properties *in vitro* and *in vivo* . J. Cell Mol. Med. 22 (3), 1428–1442. 10.1111/jcmm.13492 29392844 PMC5824372

[B23] Ishiuchi N. Nakashima A. Doi S. Yoshida K. Maeda S. Kanai R. (2020). Hypoxia-preconditioned mesenchymal stem cells prevent renal fibrosis and inflammation in ischemia-reperfusion rats. Stem Cell Res. Ther. 11 (1), 130. 10.1186/s13287-020-01642-6 32197638 PMC7083035

[B24] Jafarinia M. Alsahebfosoul F. Salehi H. Eskandari N. Ganjalikhani-Hakemi M. (2020). Mesenchymal stem cell-derived extracellular vesicles: a novel cell-free therapy. Immunol. Invest. 49 (7), 758–780. 10.1080/08820139.2020.1712416 32009478

[B25] Jeong J. O. Han J. W. Kim J. M. Cho H. J. Park C. Lee N. (2011). Malignant tumor formation after transplantation of short-term cultured bone marrow mesenchymal stem cells in experimental myocardial infarction and diabetic neuropathy. Circ. Res. 108 (11), 1340–1347. 10.1161/CIRCRESAHA.110.239848 21493893 PMC3109741

[B26] Jin Y. Ma L. Zhang W. Yang W. Feng Q. Wang H. (2022). Extracellular signals regulate the biogenesis of extracellular vesicles. Biol. Res. 55 (1), 35. 10.1186/s40659-022-00405-2 36435789 PMC9701380

[B27] Kany S. Vollrath J. T. Relja B. (2019). Cytokines in inflammatory disease. Int. J. Mol. Sci. 20 (23), 6008. 10.3390/ijms20236008 31795299 PMC6929211

[B28] Karp J. M. Leng Teo G. S. (2009). Mesenchymal stem cell homing: the devil is in the details. Cell Stem Cell 4 (3), 206–216. 10.1016/j.stem.2009.02.001 19265660

[B29] Kou M. Huang L. Yang J. Chiang Z. Chen S. Liu J. (2022). Mesenchymal stem cell-derived extracellular vesicles for immunomodulation and regeneration: a next generation therapeutic tool? Cell Death Dis. 13 (7), 580. 10.1038/s41419-022-05034-x 35787632 PMC9252569

[B30] Kusuma G. D. Carthew J. Lim R. Frith J. E. (2017). Effect of the microenvironment on mesenchymal stem cell paracrine signaling: opportunities to engineer the therapeutic effect. Stem Cells Dev. 26 (9), 617–631. 10.1089/scd.2016.0349 28186467

[B31] Lataillade J. Doucet C. Bey E. Carsin H. Huet C. Clairand I. (2007). New approach to radiation burn treatment by dosimetry-guided surgery combined with autologous mesenchymal stem cell therapy. Regen. Med. 2 (5), 785–794. 10.2217/17460751.2.5.785 17907931

[B32] Le Blanc K. Frassoni F. Ball L. Locatelli F. Roelofs H. Lewis I. (2008). Mesenchymal stem cells for treatment of steroid-resistant, severe, acute graft-versus-host disease: a phase II study. Lancet 371 (9624), 1579–1586. 10.1016/S0140-6736(08)60690-X 18468541

[B33] Li A. Dubey S. Varney M. L. Dave B. J. Singh R. K. (2003b). IL-8 directly enhanced endothelial cell survival, proliferation, and matrix metalloproteinases production and regulated angiogenesis. J. Immunol. 170 (6), 3369–3376. 10.4049/jimmunol.170.6.3369 12626597

[B34] Li J. Zhang Y. P. Kirsner R. S. (2003a). Angiogenesis in wound repair: angiogenic growth factors and the extracellular matrix. Microsc. Res. Tech. 60 (1), 107–114. 10.1002/jemt.10249 12500267

[B35] Liu Y. J. Wang C. (2023). A review of the regulatory mechanisms of extracellular vesicles-mediated intercellular communication. Cell Commun. Signal 21 (1), 77. 10.1186/s12964-023-01103-6 37055761 PMC10100201

[B36] Milbank E. Dragano N. R. V. González-García I. Garcia M. R. Rivas-Limeres V. Perdomo L. (2021). Small extracellular vesicle-mediated targeting of hypothalamic AMPKα1 corrects obesity through BAT activation. Nat. Metab. 3 (10), 1415–1431. 10.1038/s42255-021-00467-8 34675439

[B37] Neuhuber B. Swanger S. A. Howard L. Mackay A. Fischer I. (2008). Effects of plating density and culture time on bone marrow stromal cell characteristics. Exp. Hematol. 36 (9), 1176–1185. 10.1016/j.exphem.2008.03.019 18495329 PMC2603339

[B38] Phinney D. G. Pittenger M. F. (2017). Concise review: MSC-derived exosomes for cell-free therapy. Stem Cells 35 (4), 851–858. 10.1002/stem.2575 28294454

[B39] Samsonraj R. M. Raghunath M. Nurcombe V. Hui J. H. van Wijnen A. J. Cool S. M. (2017). Concise review: multifaceted characterization of human mesenchymal stem cells for use in regenerative medicine. Stem Cells Transl. Med. 6 (12), 2173–2185. 10.1002/sctm.17-0129 29076267 PMC5702523

[B40] Sarsenova M. Kim Y. Raziyeva K. Kazybay B. Ogay V. Saparov A. (2022). Recent advances to enhance the immunomodulatory potential of mesenchymal stem cells. Front. Immunol. 13, 13. 10.3389/fimmu.2022.1010399 PMC9537745 36211399

[B41] Sohni A. Verfaillie C. M. (2013). Mesenchymal stem cells migration homing and tracking. Stem Cells Int. 2013, 1–8. 10.1155/2013/130763 PMC3806396 24194766

[B42] Sung D. K. Chang Y. S. Sung S. I. Ahn S. Y. Park W. S. (2019). Thrombin preconditioning of extracellular vesicles derived from mesenchymal stem cells accelerates cutaneous wound healing by boosting their biogenesis and enriching cargo content. J. Clin. Med. 8 (4), 533. 10.3390/jcm8040533 31003433 PMC6517934

[B43] Théry C. Witwer K. W. Aikawa E. Alcaraz M. J. Anderson J. D. Andriantsitohaina R. (2018). Minimal information for studies of extracellular vesicles 2018 (MISEV2018): a position statement of the International Society for Extracellular Vesicles and update of the MISEV2014 guidelines. J. Extracell. Vesicles 7 (1), 1535750. 10.1080/20013078.2018.1535750 30637094 PMC6322352

[B44] Uccelli A. Moretta L. Pistoia V. (2008). Mesenchymal stem cells in health and disease. Nat. Rev. Immunol. 8 (9), 726–736. 10.1038/nri2395 19172693

[B45] Ulpiano C. da Silva C. L. Monteiro G. A. (2021). Mesenchymal stromal cells (MSCs): a promising tool for cell-based angiogenic therapy. Curr. Gene Ther. 21 (5), 382–405. 10.2174/1566523221666210917114353 34533444

[B46] Vasanthan J. Gurusamy N. Rajasingh S. Sigamani V. Kirankumar S. Thomas E. L. (2020). Role of human mesenchymal stem cells in regenerative therapy. Cells 10 (1), 54. 10.3390/cells10010054 33396426 PMC7823630

[B47] Viswanathan S. Shi Y. Galipeau J. Krampera M. Leblanc K. Martin I. (2019). Mesenchymal stem versus stromal cells: international society for cell and Gene therapy (ISCT®) mesenchymal stromal cell committee position statement on nomenclature. Cytotherapy 21 (10), 1019–1024. 10.1016/j.jcyt.2019.08.002 31526643

[B48] Wang M. Yuan Q. Xie L. (2018). Mesenchymal stem cell-based immunomodulation: properties and clinical application. Stem Cells Int. 2018, 3057624–3057712. 10.1155/2018/3057624 30013600 PMC6022321

[B49] Wang S. Xu M. Li F. Wang X. Bower K. A. Frank J. A. (2012). Ethanol promotes mammary tumor growth and angiogenesis: the involvement of chemoattractant factor MCP-1. Breast Cancer Res. Treat. 133 (3), 1037–1048. 10.1007/s10549-011-1902-7 22160640 PMC3323664

[B50] Watanabe Y. Fukuda T. Hayashi C. Nakao Y. Toyoda M. Kawakami K. (2022). Extracellular vesicles derived from GMSCs stimulated with TNF-α and IFN-α promote M2 macrophage polarization via enhanced CD73 and CD5L expression. Sci. Rep. 12 (1), 13344. 10.1038/s41598-022-17692-0 35922474 PMC9349189

[B51] Witwer K. W. Goberdhan D. C. O’Driscoll L. Théry C. Welsh J. A. Blenkiron C. (2021). Updating MISEV: evolving the minimal requirements for studies of extracellular vesicles. J. Extracell. Vesicles 10 (14), 10. 10.1002/jev2.12182 PMC8710080 34953156

[B52] Xunian Z. Kalluri R. (2020). Biology and therapeutic potential of mesenchymal stem cell‐derived exosomes. Cancer Sci. 111 (9), 3100–3110. 10.1111/cas.14563 32639675 PMC7469857

[B53] Zhuang W. Z. Lin Y. H. Su L. J. Wu M. S. Jeng H. Y. Chang H. C. (2021). Mesenchymal stem/stromal cell-based therapy: mechanism, systemic safety and biodistribution for precision clinical applications. J. Biomed. Sci. 28 (1), 28. 10.1186/s12929-021-00725-7 33849537 PMC8043779

